# Imaging Characteristics of Neovascular and Atrophic Pachychoroidal Spectrum Diseases

**DOI:** 10.3389/fmed.2022.891397

**Published:** 2022-07-04

**Authors:** Rui Hua, Meixia Zhang

**Affiliations:** ^1^Department of Ophthalmology, First Hospital of China Medical University, Shenyang, China; ^2^Department of Ophthalmology, West China Hospital, Sichuan University, Chengdu, China; ^3^Research Laboratory of Macular Disease, West China Hospital, Sichuan University, Chengdu, China

**Keywords:** pachychoroid spectrum diseases, optical coherence tomography angiography, choroidal vascular index, atrophic, aneurysmal polypoidal lesions

## Abstract

**Background:**

This study qualitatively and quantitatively compared imaging characteristics between neovascular and atrophic pachychoroid spectrum disease (PSD) by optical coherence tomography (OCT), and OCT angiography (OCTA).

**Methods:**

The subtypes of PSD were identified by multi-modality imaging approaches. Subfoveal choroidal thickness (SFCT), choroidal vascular index (CVI), and vascular density of choroidal neovascularization (CNV) were measured.

**Results:**

The CVI and SFCT of 174 PSD eyes were 67.6% ± 5.48% and 362.2 ± 131.88 μm, respectively. After adjustment for age, linear regression indicated that SFCT was positively associated with CVI (*p* < 0.001), and patched hyper-reflective lesions in choriocapillaris layers (*p* = 0.009). Compared with neovascular PSD eyes, atrophic PSD eyes had similar patient age (57.1 ± 16.72 years, *p* = 0.639), SFCT (332.0 ± 111.00 μm, *p* = 0.51), and CVI (67.6% ± 3.94%, *p* = 0.527). There were no differences between polypoidal choroidal vasculopathy (PCV) eyes with aneurysmal polypoidal lesions and PCV eyes with tangled polypoidal lesions in terms of age, CVI, SFCT, vascular density, or the occurrence of double layer signs (DLSs, all *p* > 0.05). Logistic regression indicated that age (*p* = 0.003), SFCT (*p* = 0.003), patched hyper-reflective lesions in choriocapillaris layers (*p* = 0.009), and DLSs (*p* < 0.001) were predictive factors for CNV progression in PSD eyes (all *p* < 0.05).

**Conclusions:**

Our study highlighted the similarities in SFCT and CVI between neovascular and atrophic PSD, both of which were late stage lesions. Besides, age, SFCT, patched hyper-reflective lesions in choriocapillaris layers, and DLSs were risk factors for CNV in PSD. Our results showed that atrophic PSD is an important change in the late stage of PSD disease, which is helpful for in-depth understanding of the pathological mechanism of PSD and corresponding intervention.

## Background

In 2013, Warrow et al. (1) introduced the term “pachychoroid” to identify a set of macular disorders that were generally characterized by a subfoveal choroidal thickness (SFCT) of >300 μm. Recently, Siedlecki et al. suggested regrouping pachychoroid spectrum disease (PSD) into five successive types: uncomplicated pachychoroid (UCP, type 0), pachychoroid pigment epitheliopathy (PPE, type I), central serous chorioretinopathy (CSC, type II), pachychoroid neovasculopathy (PNV, type III) with two sub-phenotypes (IIIa involves neurosensory detachment and overlaps with CSC; IIIb does not involve neurosensory detachment), and aneurysmal type 1 neovascularization/polypoidal choroidal vasculopathy (PCV, type IV) (2). Moreover, in early 2019, Cheung et al. included focal peripapillary pachychoroid syndrome (PPS) and focal choroidal excavation (FCE) in PSD (3). The common pathological characteristics were dilated veins (pachyvessels) in the Haller's layer with choroidal vascular hyperpermeability, combined with thinning of the choriocapillaris and Sattler's layers (4).

In addition to neovascular lesions in PNV and PCV, atrophic lesions have been reported in PSD. For example, pachychoroid geographic atrophy (GA) is associated with choriocapillaris obliteration (5). Furthermore, atrophy of the retinal pigment epithelium (RPE), choriocapillaris, and choroidal stroma is a major characteristic of PSD pathogenesis. With CSC progression, RPE atrophy occurs after damage to photoreceptor cells, which manifests as typical decreased blue light autofluorescence (BL-AF) (6). The absence of choriocapillaris optical coherence tomography (OCT) angiography (OCTA) changes in altered RPE adjacent to atrophic regions suggests that choriocapillaris injury is not present (7). Choroidal caverns in PSD are presumed to originate from nonperfused vessels (8). Notably, 52% of pachychoroid eyes exhibit choroidal lipid globule caverns (9).

The formation of FCE involves choroidal ischemia because of the underlying contraction of scarred choroidal connective tissue from previous/subclinical inflammatory processes, as well as focal choroidal atrophy (10). Moreover, some atrophic PSD can progress into neovascular PSD, such as choroidal neovascularization (CNV) secondary to both FCE and chronic CSC.

Age-related macular degeneration (AMD) is known to progress from early AMD to intermediate and late forms of AMD, which include neovascular AMD and GA (11). We presume that atrophy progression is an important manifestation in the late stage of PSD and may comprise its typical pathogenesis. To our knowledge, the imaging characteristics of atrophic PSD, as well as its differences from neovascular PSD, have not been comprehensively analyzed thus far. Here, we qualitatively and quantitatively compared imaging characteristics between neovascular and atrophic PSD. We also examined the activity of CNV in PSD and the types of polypoidal lesions by OCTA, in order to further explore the predictors of CNV in PSD affected eyes, and provide guidance for understanding the pathological mechanism and treatment strategy of PSD.

## Methods

This retrospective, hospital-based study adhered to the tenets of the Declaration of Helsinki, and was reviewed and approved by the Institutional Review Board of the First Hospital of China Medical University, Shenyang, China (No. AF-OG-03-1.1-02). Written informed consent were obtained from all patients for their medical information to be included in our study.

### Study Participants

The PSD inclusion criteria were thinning of the choriocapillaris and Sattler's layer, and the presence of pachyvessels/dilated choroidal vessels in Haller's layer in B-scan/en face OCT, or OCTA; patients were included regardless of RPE abnormalities overlying the pachyvessels (12). The identification of PSD subtypes was performed in accordance with the methods used by Siedlecki et al. (2) and Cheung et al. (3). PCV diagnostic criteria were established using the 2005 Japanese Study Group guidelines (13), based on the characteristic polypoidal lesions at the border of the choroidal branching vascular networks (BVNs) in indocyanine green angiography (ICGA) (14). In addition, we also checked characteristic polyps with BVNs in B-scan OCTA (15) or en face OCTA (16). OCTA visualized polypoidal lesions as ring-like flow signals/incomplete round/ring-like flow signals overlaid with ring-like OCT structures inside RPE detachments, or flow signals adjacent to a RPE detachment notch (15), or tangled vascular structures with BVNs or type 2 CNV (16). Patients with diabetic retinopathy, retinal vein occlusion, uveitis, glaucoma, and the patient who had received fundus laser therapy, vitrectomy, or anti- vascular endothelial growth factor (VEGF) therapy, in either eye were excluded. Moreover, systematic diseases, such as metabolic disorders and hypertension, were also excluded from this study.

### Examinations and Parameter Measurements

The following multi-modality imaging approaches were used to identify all PSD related lesions: OCTA (Spectralis, Heidelberg Engineering, Heidelberg, Germany; SSADA-OCTA, Optovue OCT, Optovue, Inc, Fremont, California, USA), OCT (Spectralis, Heidelberg Engineering; SSADA-OCT, Optovue OCT, Optovue), BL-AF (Spectralis HRA+OCT; Heidelberg Engineering), fundus fluorescein angiography (FFA, Spectralis HRA+OCT, Heidelberg Engineering), and ICGA (Spectralis HRA+OCT, Heidelberg Engineering).

Pachychoroid spectrum disease with CNV was regarded as neovascular PSD; PSD without CNV and atrophy lesions was regarded as control group; non-neovascular PSD with atrophy lesions (e.g., RPE atrophy secondary to CSC, simple RPE atrophy, pachychoroid GA, FCE, and choroidal lipid globule caverns) was regarded as atrophic PSD. Especially, GA was defined as sharply demarcated atrophic lesions of the outer retina, initially sparing the foveal center, then gradually expand and coalesce to include the fovea (17). En face OCT rendered choroidal lipid globule caverns as sharply delimited hyporeflective areas, primarily within inner choroidal stroma, similar to the size of choroidal vessels, with typical posterior hypertransmission in OCT and without flow signal in OCTA (18).

Pachychoroid neovasculopathy was divided into IIIa, IIIb, and IIIc types (PSD with type 2 CNV), as in our previous study (19). The PCV polypoidal flow was classified into aneurysmal, and tangled (16) according to their signal structures in OCTA. Moreover, the CNV activities in PSD in OCTA (e.g., PNV, BVNs in PCV, and CNV secondary to FCE) were graded as active, and inactive, in accordance with the method used by Coscas et al. (20), and a lesion was assessed to have all or at least three of the following five features: firstly, a lacy-wheel or sea-fan shaped CNV lesion; secondly, branching, numerous tiny capillaries; thirdly, the presence of loops and anastomoses; and then, the presence of a peripheral arcade; finally, the presence of a perilesional hypointense halo.

**Figure 1:**
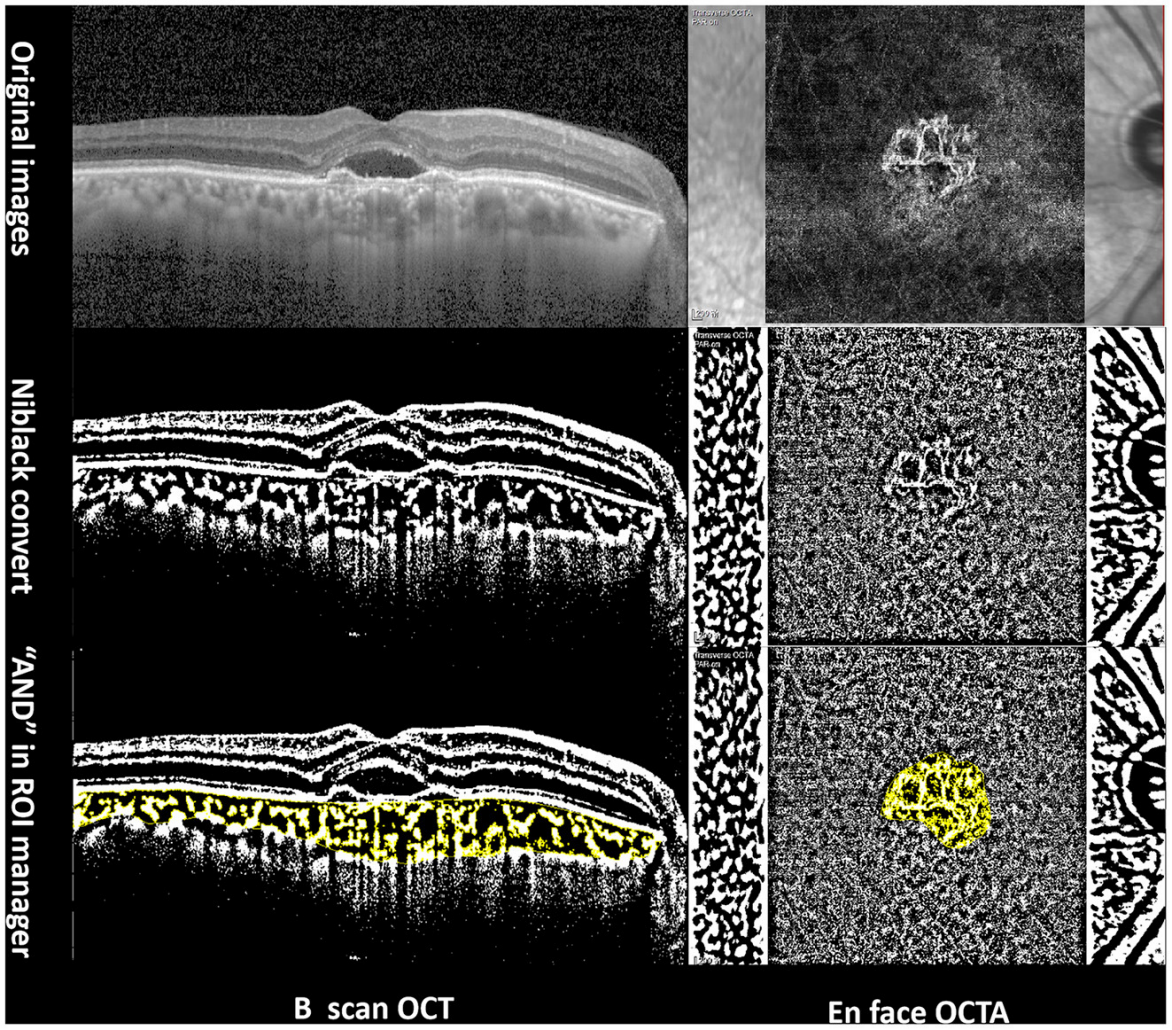
Imaging analysis of a 65-year-old PCV patient using Image J software. Left column: choroidal vascular index in B-scan OCT. Right column: vascular density measurement of CNV in OCTA.

Image processing, choroidal vascular index (CVI) measurements in B-scan OCT (Figure 1), and vascular density (VD) of CNV measurements in OCTA (Figure 1) were conducted using the Niblack threshold method in Image J software (version 1.52, National Institutes of Health, Bethesda, MD, USA), in accordance with the method described by Wei et al. (21). CVI was measured in one section of 30-degree B-scan foveal OCT. VD was defined as the percentage area of vasculature (pixels) above the threshold level (22).

### Statistical Analyses

All data were analyzed by SPSS Statistics (version 24.0, SPSS, Inc., Chicago, IL, USA), and presented as means ± standard deviations for variables with normal distributions. Single-factor repeated measures analysis of variance (ANOVA) with *post hoc* LSD test, Fisher's exact test, Pearson's test, independent-samples *t*-test, and linear and multiple logistic regression were performed. We also calculated odds ratios (ORs) and 95% confidence intervals (CIs). *p*-value < 0.05, was defined as statistical significance.

## Results

### Demographic Information

**Figure 2:**
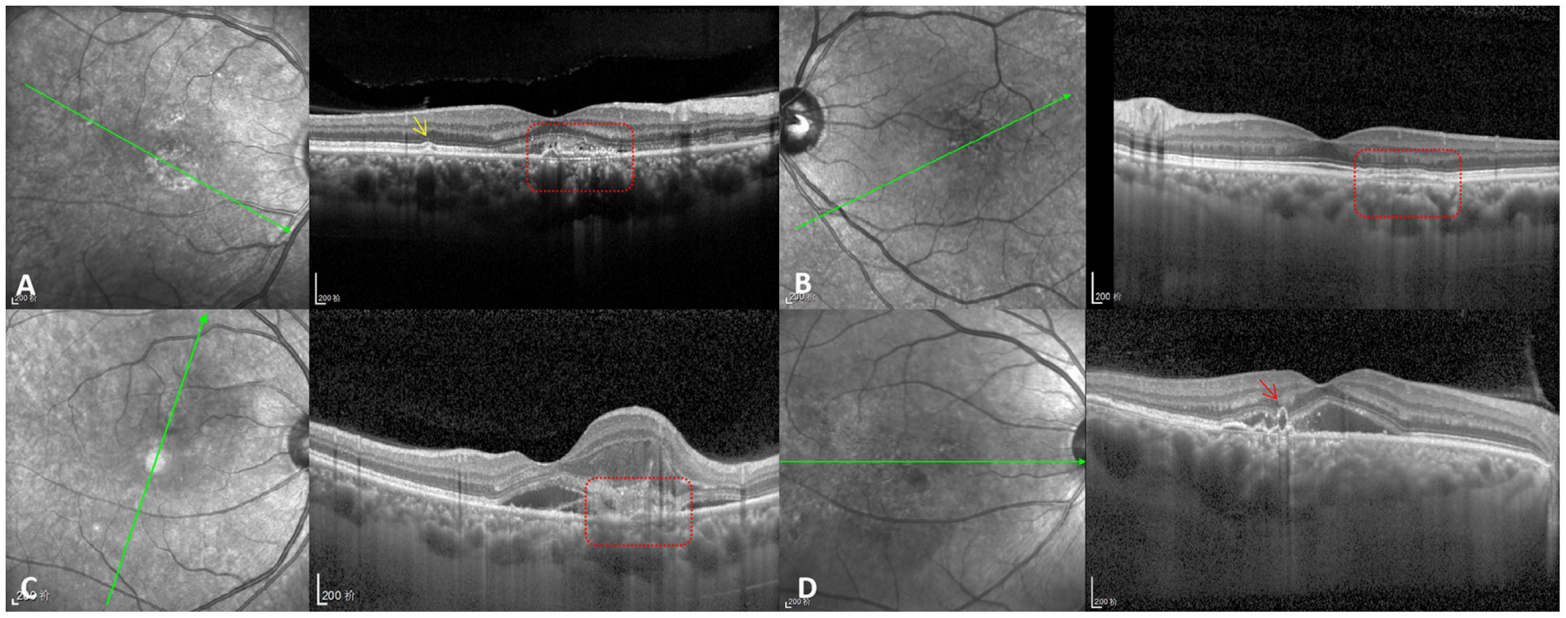
CNV types in PSD patients. **(A)** Type IIIa PNV (red dotted box) with pachydrusen (yellow arrow). **(B)** Type IIIb PNV (red dotted box). **(C)** Type IIIc PNV (red dotted box) with type 2 CNV. **(D)** Naked polypoidal lesion (red arrow) in a PCV eye.

This study enrolled 131 PSD patients with the mean age of 54.1 ± 12.27 years (Table 1, Figure 2). The mean patient age of CSC eyes was 47.9 ± 11.47 years, which was significantly younger than that in PNV eyes (53.1 ± 12.84 years, *p* = 0.044) and PCV eyes (62.5 ± 7.14 years, *p* < 0.001). Moreover, the mean patient age of PNV eyes was significantly younger than that of PCV eyes (*p* = 0.001).

**Table 1:** Demographic information.

**131 PSD patients (174 eyes)**							
Gender	83 men; 48 women						
Mean age	54.1 ± 12.27 years						
Subtypes	16 UCP eyes (9.2%)	24 PPE eyes (13.8%)	2 PPS eyes (1.1%)	11 FCE eyes (6.3%; one eye with nonconforming FCE and 10 eyes with conforming FCE)	47 CSC eyes (27.0%)	27 PNV eyes (15.5%; 12 type IIIa eyes, 10 type IIIb eyes, and five type IIIc eyes)	36 PCV eyes (20.7%)

The SFCT and CVI of 174 PSD eyes were 362.2 ± 131.88 μm and 67.6% ± 5.48%, respectively. The SFCT of CSC eyes was 442.1 ± 120.80 μm, which was significantly greater than that in PNV eyes (343.8 ± 124.30 μm, *p* = 0.001) and PCV eyes (285.4 ± 125.80 μm, *p* < 0.001). However, there was no difference in SFCT between PNV and PCV eyes (*p* = 0.066). The CVI of CSC eyes was 69.2% ± 6.11%, which was significantly greater than the CVI of PNV eyes (66.1% ± 2.90%, *p* = 0.028) and comparable with the CVI of PCV eyes (67.2% ± 6.80%, *p* = 0.127). There was no difference of CVI between PNV and PCV (*p* = 0.438).

**Figure 3:**
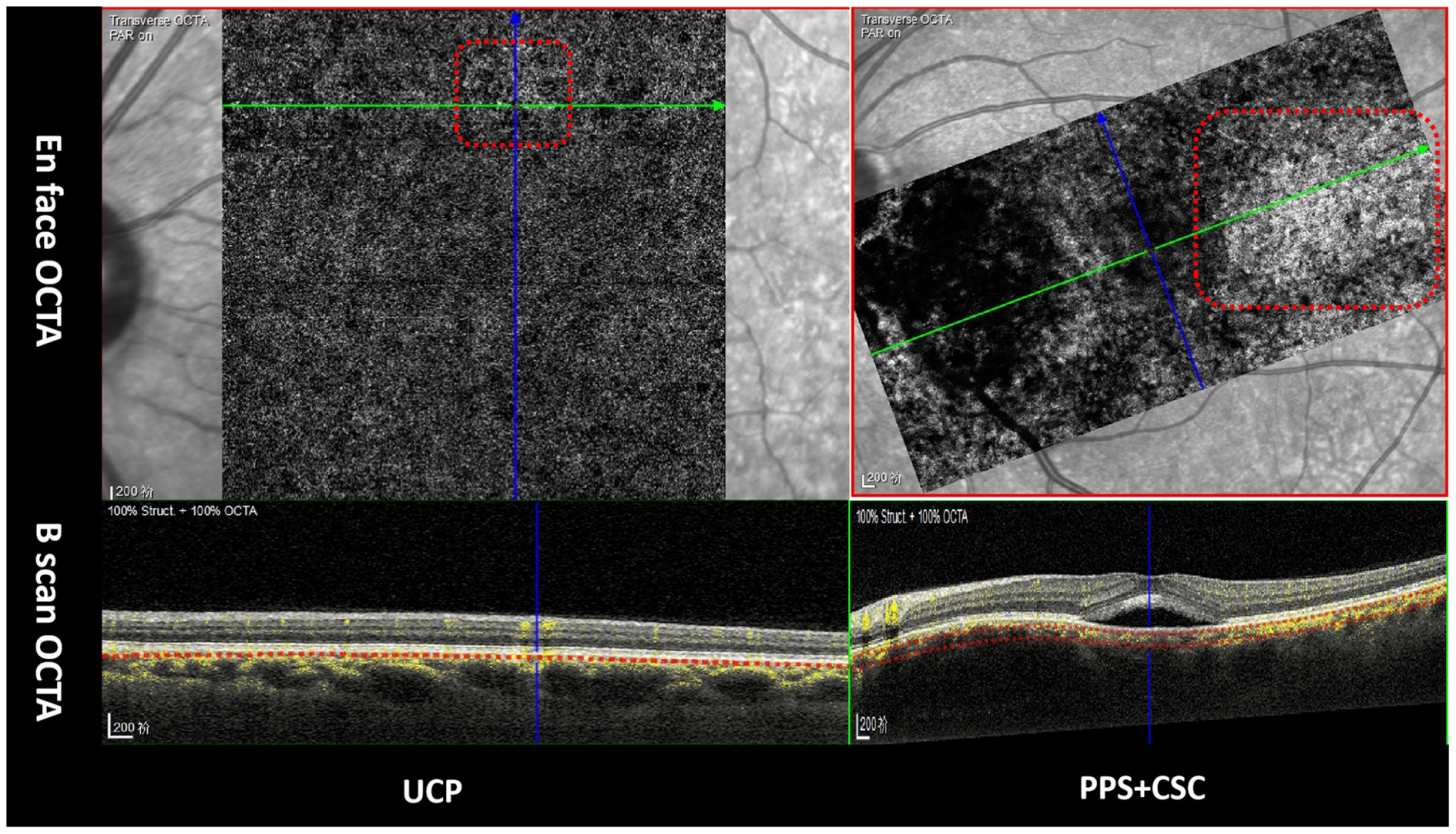
Patched hyper-reflective lesions in choriocapillaris layers in OCTA (red dotted box). Green arrows in upper line indicate the orientation of B-scan OCTA in lower line.

In total, 24 eyes (13.8%) showed patched hyper-reflective lesions in choriocapillaris layers during OCTA examination (Figure 3). SFCT was positively correlated with CVI (*r* = 0.279, *p* < 0.001) and patched hyper-reflective lesions (*r* = 0.244, *p* = 0.001), and negatively correlated with age (*r* = −0.33, *p* < 0.001). However, no correlation was found between CVI and age (*r* = 0.021, *p* = 0.784). Double layer signs (DLSs) of RPE were detected in 78 eyes (44.8%) by OCT, which were not correlated with CVI (*r* = 0.005, *p* = 0.948) or SFCT (*r* = 0.111, *p* = 0.143). After adjustment for age, linear regression indicated that SFCT was positively associated with CVI (*t* = 3.801, *p* < 0.001; 95%CI: 305.68–966.29), and patched hyper-reflective lesions (*t* = 2.656, *p* = 0.009; 95%CI: 17.75–120.48).

**Figure 4:**
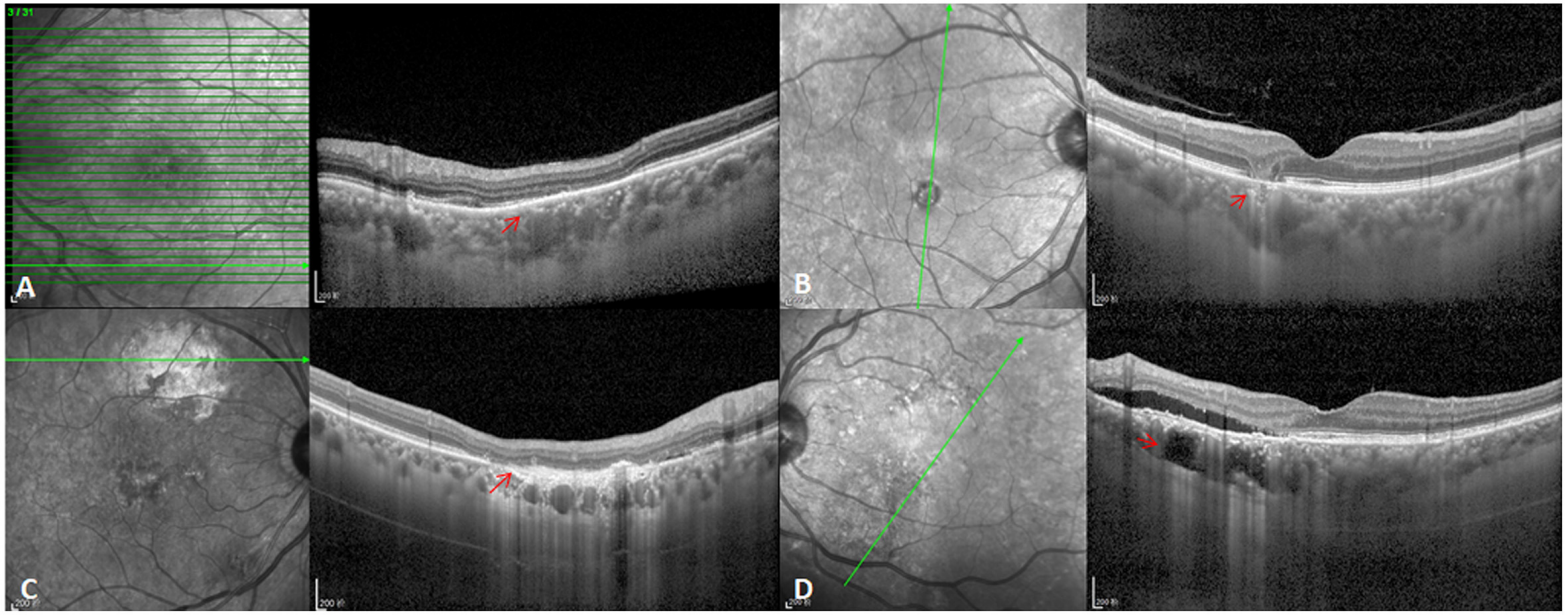
Atrophic lesion types in PSD eyes. **(A)** CSC-related RPE atrophy lesions (red arrow). **(B)** GA lesions (red arrow). **(C)** Simple RPE atrophy (red arrow). **(D)** Choroidal lipid globulecavern (red arrow).

Besides, five eyes had GA lesions, three eyes had CSC-related RPE atrophy lesions, two eyes had simple RPE atrophy, and five eyes had choroidal lipid globule cavern (Figure 4). Moreover, 22 eyes had pachydrusens.

### Neovascular PSD and Non-CNV PSD

Optical Coherence Tomography showed that 111 PSD eyes did not have CNV and 63 PSD eyes had neovascular PSD (56 with type 1 CNV, six with type 2 CNV, and one with mixed type CNV). The VD of CNV in PSD eyes was 38.3% ± 3.30%. The VD of PNV eyes was 39.4% ± 3.18%, which was significantly greater than that of PCV eyes (37.5% ± 3.19%, *t* =2.361, *p* = 0.021). OCTA examination of the 63 CNV eyes revealed active CNV in 51 eyes and inactive CNV in 12 eyes. No differences were observed between active and inactive CNV eyes in terms of age, SFCT, CVI, or VD (Table 2).

**Table 2:** The comparison of influencing parameters.

	**Age (years)**	**SFCT (μm)**	**CVI (%)**	**VD (%)**	**DLSs**
51 active CNV eyes	58.1 ± 11.20	305.6 ± 120.21	66.2 ± 4.54	38.5 ± 3.54	
12 inactive CNV eyes	60.0 ± 10.07(*t* = 0.528, *p* = 0.6)	331.3 ± 159.35 (*t* = 0.625, *p* = 0.534)	68.8 ± 8.31 (*t* = 1.48, *p* = 0.144)	37.4 ± 1.86 (*t* = 1.043, *p* = 0.301)	
19 atrophy PSD eyes	57.1 ± 16.72	332.0 ± 111.00	67.6 ± 3.94		
92 control group eyes	50.4 ± 10.98 (Compared with atrophy PSD: *p* = 0.026)	403.9 ± 125.47(compared with atrophy PSD: *p* = 0.023)	68.1 ± 5.51 (compared with atrophy PSD: *p* = 0.684)		
63 neovascular PSD eyes	58.5 ± 10.94 (Compared with control group: *p* < 0.001; Compared with atrophy PSD: *p* = 0.639)	310.4 ± 127.52 (Compared with control group: *p* < 0.001; Compared with atrophy PSD: *p* = 0.51)	66.7 ± 5.47 (Compared with control group: *p* = 0.103; Compared with atrophy PSD: *p* = 0.527)		
23 PCV eyes with aneurysmal polypoidal lesions	63.1 ± 7.59	281.1 ± 123.33	67.5 ± 7.25	37.5 ± 3.20	18 eyes
7 PCV eyes with tangled polypoidal lesions	61.4 ± 6.35 (*t* = 0.537, *p* = 0.595)	250.3 ± 116.62 (*t* = 0.584, *p* = 0.564)	68.4 ± 3.57 (*t* = 0.29, *p* = 0.774)	36.5 ± 3.60 (*t* = 0.742, *p* = 0.464)	5 eyes (Fisher *p* = 0.532)

The mean patient age was significantly younger in the non-CNV PSD eyes than in the neovascular PSD eyes (51.5 ± 12.32 years vs. 58.5 ± 10.94 years, *t* =3.717, *p* < 0.001). CVI was similar in PSD eyes with and without CNV (66.7% ± 5.47% vs. 68.1% ± 5.27%, *t* =1.594, *p* = 0.113). In contrast, SFCT was significantly greater in PSD eyes without CNV than in neovascular PSD eyes (391.6 ± 125.62 μm vs. 310.4 ± 127.52 μm, *t* = 4.075, *p* < 0.001).

Among 111 PSD eyes without CNV, 20 had pachydrusen, 22 had patched hyper-reflective lesions in choriocapillaris layers, and 32 had DLSs. Among 63 neovascular PSD eyes, none had pachydrusen, two had patched hyper-reflective lesions in choriocapillaris layers, and 46 had DLSs. The proportions of eyes with pachydrusen and patched hyper-reflective lesions in choriocapillaris layers were significantly greater in PSD eyes without CNV than in neovascular PSD eyes (Fisher *p* < 0.001). In contrast, the proportion of eyes with DLSs was significantly lower in PSD eyes without CNV than in neovascular PSD eyes (Fisher *p* < 0.001).

### Neovascular PSD and Atrophy PSD

Among 111 PSD eyes without CNV, 19 had atrophic PSD, and the left 92 PSD eyes were enrolled into control group. The mean patient age of control group was significantly younger than that of atrophy PSD eyes (*p* = 0.026), and neovascular PSD eyes (*p* < 0.001). However, the latter two failed to show any difference in age (*p* = 0.639). Additionally, the SFCT in control group was significantly thicker than that in both atrophy PSD eyes (*p* = 0.023), and neovascular PSD eyes (*p* < 0.001). However, the latter two failed to show any difference in SFCT (*p* = 0.51). There was also no difference of CVI among atrophic PSD eyes, neovascular PSD eyes and control group (Table 2).

Logistic regression revealed that age (*B* = 0.047, *p* = 0.003; 95%CI: 1.012–1.087), SFCT (*B* = −0.006, *p* = 0.003; 95%CI: 0.991–0.998), patched hyper-reflective lesions in choriocapillaris layers (*B* = −2.324, *p* = 0.009; 95%CI: 0.017–0.560), and DLSs (*B* = 2.648, *p* < 0.001; 95%CI: 5.766–34.625) were predictive factors for CNV progression in PSD eyes.

### Polypoidal Lesions in PCV

Optical coherence tomography showed that 31 eyes had polypoidal lesions underlying RPE detachment and four eyes had naked polypoidal lesions (Figure 2); this method missed one eye with a polypoidal lesion. Furthermore, OCTA showed that 23 eyes had aneurysmal polypoidal lesions and seven eyes had tangled polypoidal lesions; this method missed six eyes with polypoidal lesions. There was no difference between OCT (97.2%) and OCTA (83.3%) in the rate of polypoidal lesion detection (Fisher *p* = 0.053). Moreover, there were no differences between PCV eyes with aneurysmal polypoidal lesions and PCV eyes with tangled polypoidal lesions in terms of age, CVI, SFCT, VD, or the occurrence of DLSs (Table 2).

Of 23 eyes with aneurysmal polypoidal lesions, OCT showed that 21 had polypoidal lesions underlying RPE detachment (91.3%) and the remaining two had naked polypoidal lesions (8.7%). Additionally, OCT examination of seven eyes with tangled polypoidal lesions showed that six had polypoidal lesions underlying RPE detachment (85.7%) and the remaining one had naked polypoidal lesions (14.3%). However, there was no difference between these two groups in the occurrence of naked polypoidal lesions (Fisher *p* = 0.254). OCTA examination of 23 eyes with aneurysmal polypoidal lesions revealed that 17 had active CNV (73.9%), which was similar to the number of eyes with active CNV (seven) among seven eyes with tangled polypoidal lesions (100%, Fisher *p* = 0.170).

## Discussion

In our study, we analyzed the imaging characteristics of 174 PSD eyes in 131 patients. Thus far, no epidemiologic data regarding PSD have been published. To our knowledge, this is the first study to compare characteristics (e.g., SFCT, CVI, and VD) between atrophic and neovascular patterns; to investigate the activity of CNV in PSD; and to use OCTA to examine the types of polypoidal lesions (e.g., aneurysmal, and tangled).

Notably, 13.8% of eyes showed patched hyper-reflective lesions in choriocapillaris layers in OCTA in our study. Previously, Lee et al. (23) reported attenuation of the inner choroidal layer on a cross-sectional B-scan, secondary to the dilatation of outer choroidal vessels, which forms inner choroidal obscuration. CSC related choroidal exudations may present as hyporeflective regions from inner to outer choroidal layer. In our study, we observed patched hyper-reflective lesions caused by the dilatation of choroidal vessels in Haller's layer, which resulted in congested choriocapillaris in the superior level of choriocapillaris layers, in contrast to hyporeflective reflex of inner choroidal obscuration (23).

The universal mean values for SFCT and CVI in our study were slightly greater than the corresponding values in healthy eyes (SFCT, 306.05 ± 55.34 μm; CVI, 66.71% ± 2.58%) (24). In contrast, Lee et al. (25) reported that the mean SFCTs of PSD were 428.5 ± 57.9 μm and 430.5 ± 68.1 μm using both spectral domain and swept source (SS) OCT, respectively, which were greater than the findings in our study. Previously, Lehmann et al. (26), reported that 395 μm was the limit of normal SFCT, based on sensitivity and specificity values of 76.4 and 60%, respectively. However, multiple factors influence SFCT, and no definitive quantitative standard for PSD has been established until now. Moreover, our results showed that SFCT had a positive relationship with CVI and patched hyper-reflective lesions, whereas it was negatively correlated with age. Because CVI is reportedly significantly correlated with pachyvessels (27), both the diameter (28) and area of choroidal hyporeflective lumina considerably contribute to SFCT, whereas choroidal stroma do not (29). Koçak et al. (24) reported that SFCT was significantly greater in participants aged ≤18 years than in participants aged >18 years. However, our participants were considerably older; this increased age may have led to stromal atrophy and vascular arteriosclerosis in the choroid, thus resulting in lower SFCT. Furthermore, CVI was reportedly significantly greater in participants aged ≤ 18 years than in participants aged >18 years (24). However, we found no relationship between CVI and age. Multiple factors influence CVI, including PSD status and subtypes. Ng et al. (27), found that exudative maculopathy with pachyvessels was associated with younger age (69.1 ± 9.4 years), increased SFCT, and increased CVI (65.4% ± 5.3%). Our participants were younger, but their CVI values were similar.

In our study, OCT revealed DLSs of RPE in 78 eyes. DLSs on B-scan OCT have been associated with preclinical type 1 macular neovascularization with good predictive values in non-exudative AMD eyes (30). Additionally, Yang et al. (28) found that DLSs occurred significantly more often in chronic CSC eyes than in acute CSC eyes. In our previous study, we suggested that an irregular near infrared pattern, together with DLSs, was significantly positively correlated with the occurrence of choroidal vascular diffuse hyperpermeability (31). However, in the present study, DLSs were not associated with CVI or SFCT. Lim et al. (32) reported that SFCT is not an accurate parameter for diagnosis of pachychoroid disease; they also indicated that SFCT does not represent the entire choroidal area. Therefore, we presumed that focal enhancement of CVI and choroidal thickness cannot fully indicate PSD status; furthermore, some PSD eyes had increased choroidal thickness outside the fovea. Importantly, we previously showed that choroidal thickness at polyp sites was positively associated with polyp size, while choroidal thickness did not significantly differ between foveal and polyp sites (33); our current findings are consistent with these results.

In this study, OCT revealed four eyes with naked polypoidal lesions. Similarly, using SS-OCTA, Bo et al. (16) observed that polypoidal lesions coexisted with type 1 and 2 CNV in the same PCV eyes. The etiology of naked polypoidal lesions might involve massive exudation from hyalinized vessels (identified on histopathological examination), increased intra-tissue pressure (triggering the erosion of polypoid choroidal vessel breaks through RPE), and Bruch's membrane disruption (34).

Additionally, OCTA examination showed that 23 eyes had aneurysmal polypoidal lesions and seven eyes had tangled polypoidal lesions. Both choroidal arteriosclerosis and greater hydrostatic pressure contribute to the formation of hyalinized choroidal arteries and aneurismal dilatations, eventually resulting in PCV. Aneurysmal or polypoidal elements may represent proliferative components at the advancing edge of BVN (35). Li et al. (36) demonstrated that hyalinized choroidal arteries progress to capillary beds within the sub-RPE; a lumen with thin vessel walls can be visualized in corresponding histological sections near the aneurysmal site. Recently, Bo et al. (16) used SS-OCTA to demonstrate that previous polypoidal lesions may appear as tangled vascular structures adjacent to type 2 CNV or BVNs; we presume that the observation of “tangled PCV” without aneurysms may be a subtype of PNV or an attenuated form of PCV (19). In this study, we observed no differences between PCV eyes with aneurysmal polypoidal lesions and PCV eyes with tangled polypoidal lesions in multiple characteristics. Bo et al. (16) reported that aneurysms would presumably be unresponsive to anti-VEGF treatment, whereas a tangle of new vessels is likely to respond.

We found no difference between OCT and OCTA in the rate of polypoidal lesion detection. However, OCTA tended to have a lower detective rate, possibly because turbulent blood flow only circulates at the aneurysmal wall (37).

Chang et al. (38) reported that, compared with nonpachychoroidal PCV patients, pachychoroid PCV patients were significantly younger, with fewer AMD-like features, more CSC-like features, and less sensitive to anti-VEGF treatment. CSC, PNV, and PCV are considered signs of a continuous disease process that eventually involves choroidal malfunction (2). Notably, our study showed that the patient age was significantly lower in CSC eyes than in PNV eyes and PCV eyes; moreover, the patient age was significantly lower in PNV eyes than in PCV eyes. Age is thus important in the progressions of CSC, PNV, and PCV. Lee et al. (39) suggested that non-exudative PNV, which is frequently identified without symptoms among older patients, is a critical precursor of PCV. Arteriosclerosis in older patients can cause choriocapillaris obliteration or ischemia, as well as RPE damage (29). Age-related and hypertension-related choroidal arteriosclerosis and greater hydrostatic pressure cause the formation of hyalinized choroidal arteries and aneurysmal dilatations, eventually resulting in PCV (19). This progression is supported by the histopathologic findings of arteriosclerosis in choroidal vessels of healthy participants aged >40 years and in PCV patients (40).

Compared with PNV and PCV eyes, SFCT was significantly greater in CSC eyes. However, no difference in SFCT was observed between PNV eyes and PCV eyes, whose *p*-value of 0.066 was very close to 0.05, and we considered that small sample size (27 PNV eyes vs. 36 PCV eyes) may lead to this statistic error. Demirel et al. (41) reported that the threshold of SFCT was 422 μm for eyes with PNV and CSC, whereas it was 271 μm for eyes with PNV and PCV. SFCT was thicker in CSC eyes than in PCV or AMD eyes (42). Moreover, Demirel et al. (43) reported that the CVI of the fellow eyes was 74.93% ± 3.58% in the CSC group, which was similar to our CVI finding in CSC eyes. Furthermore, we found that the CVI was significantly greater in CSC eyes than in PNV eyes, similar to the trend regarding SFCT.

In a previous study, Lee et al. (29) found that the luminal-to-total choroidal ratio was higher in CSC eyes than in PNV eyes. Demirel et al. (41) also reported that the CVI threshold was 72.6% for eyes with PNV and PCV, whereas it was 73.6% for eyes with CSC and PCV. However, the mean CVI ratio did not significantly differ between the fellow eyes of patients with PNV and patients with CSC (43). Although PSD is a bilateral disease (19), variable results may be caused by differences among participants, PSD status, and measurement protocols. With respect to CNV development, both SFCT and CVI gradually decreased in PSD eyes, leading to choroidal atrophy. However, no available data including pathological investigation and fundus imaging analysis, has been published as a reference until now. Notably, we found that the CVI was comparable between CSC eyes and PCV eyes. In a previous study, CVI was lower in PCV eyes than in healthy eyes (44). Additionally, Bakthavatsalam et al. (45) found that the CVI was significantly lower in PCV eyes (64.94% ± 5.43%) than in healthy control eyes (68.53% ± 5.91%).

In this study, the VD was significantly greater in PNV eyes than in PCV eyes. However, there have been no other published studies concerning the VD of PNV eyes and PCV eyes. Our results indicate that PCV progression involve progression of hyalinized choroidal arteries to capillary beds within the sub-RPE; a lumen with thin vessel walls has been visualized in corresponding histological sections near the aneurysmal site (36). Thus, the CNV of PCV eyes exhibited more mature arterialization, resulting in lower VD.

In our study, five eyes had GA lesions and five eyes had choroidal lipid globule cavern lesions. Importantly, the newly described phenotype of pachychoroid GA is associated with choriocapillaris obliteration (46). However, Querques et al. (47) suggested that choroidal caverns come from nonperfused vessels. Loss of the normal choroidal architecture of dilated Haller's layer veins leads to choroidal caverns formation in PSD eyes; moreover, the loss of choroidal stroma and increased choroidal thickness facilitate increased lipid deposition (9). All of these phenotypes contribute to atrophic PSD.

Our study showed that neovascular PSD eyes had significantly older patient age than did PSD eyes without CNV. By far, age is suggested as the strongest risk factor, and for example, nearly all late AMD occur in more than 60 year old patients (48). The estimated prevalence of late AMD increased to 13.1% for more than 60 year old patients (49). Moreover, the mean patient age of atrophy PSD eyes, and neovascular PSD eyes were comparable in our study, indicating the late stage of PSD.

In this study, SFCT was significantly greater in PSD eyes without CNV than in neovascular PSD eyes. Similarly, Anna Lee, et al., reported mean SFCTs of 340.14 ± 61.59 μm (CSC), 335.17 ± 27.4 μm (thick-choroid PCV), 227 ± 42.45 μm (thin-choroid PCV), 167.58 ± 59.28 μm (AMD), and 393.77 ± 68.66 μm (PNV), which significantly differed among groups (50). The etiology may be similar to the mechanism we discussed above regarding the development of CNV: both SFCT and CVI of PSD gradually decreased, leading to choroidal atrophy. Correspondingly, our results showed that the SFCT and CVI of atrophy PSD eyes, and neovascular PSD eyes were comparable, also indicating the late stage of PSD.

Pachydrusen, composed of isolated or scattered yellow-white deposits with clear boundaries, are located in the posterior pole, beneath the RPE, associated with thick SFCT (1). Our study showed that the occurrence of pachydrusen was significantly more common in PSD eyes without CNV than in neovascular PSD eyes. To our knowledge, no research thus far has compared these two entities; Zhang et al. (51) presumed that pachydrusen constitutes a precursor of PCV and CSC. Thus, we propose that pachydrusen is an early sign of PSD, similar to variable drusen in AMD. Notably, we found that the occurrence of patched hyper-reflective lesions in choriocapillaris layers was significantly more common in PSD eyes without CNV than in neovascular PSD eyes. The main pathophysiology of pachychoroid disease includes choriocapillaris attenuation and abnormally dilated pachyvessels with choroidal vascular hyperpermeability. Additionally, choriocapillaris attenuation is accompanied by choriocapillaris hyperpermeability, obliteration, and advanced-stage ischemia (19). As we noted above, the presence of more patched hyper-reflective lesions in choriocapillaris layers in CSC eyes may indicate condensation and congestion of choriocapillaris secondary to the dilatation of outer choroidal vessels in Haller's layer. However, in PNV and PCV eyes, choriocapillaris atrophy results in less frequent occurrence of patched hyper-reflective lesions and contributes to CNV.

In contrast to the trends concerning pachydrusen and patched hyper-reflective lesions in choriocapillaris layers, the occurrence of DLSs was significantly less common in PSD eyes without CNV than in neovascular PSD eyes. Similarly, Lee et al. (52) reported that pigmentary changes, DLSs, and hypertension were independent risk factors for CNV secondary to CSC. Moreover, DLSs could serve as a manifestation or precursor lesion of PCV, reflecting the presence of fibrous tissue harbored by BVNs in PCV (53). DLSs are associated with more extensive choroidal hyperpermeability, which may occur in the presence of more severe RPE perturbation and in the inner choroidal layer; DLSs may impede metabolite transport, leading to exacerbation of outer retinal hypoxia. Thus, DLSs may indicate generally dormant inactive CNVs, with an increased risk of secondary CNV (52).

Importantly, we observed no differences in CVI between PSD eyes according to CNV status. CVI is associated with SFCT; in a previous study by Minsub Lee, no significant difference in SFCT was observed between CSC eyes and PNV eyes (29). With respect to PSD eyes without CNV, both choroidal vessels and stroma are equally involved in CSC, UCP, and PPE (54). Conversely, the choriocapillaris segment appears to be more affected in the presence of CSC, but not in the context of UCP or PPE (54). During PSD progression, we presume that choriocapillaris attenuation is accompanied by choriocapillaris hyperpermeability, obliteration, and advanced-stage ischemia (19), eventually leading to CNV and stable CVI.

A pachychoroid-driven process evokes pachychoroid GA, which shares features with PPE and PNV (55). Takahashi et al. (55) reported that after adjusting for age, pachychoroid GA patients had greater SFCT with more choroidal vascular hyperpermeability. However, no differences were found in age, SFCT, or CVI between atrophic and neovascular PSD patients.

In our study, OCT examination revealed six eyes with type 2 CNV and one eye with mixed type CNV. Either type 1 CNV (with or without polyps), type 2 CNV, or a mixed lesion can occur in FCE eyes because of choroidal ischemia (56) and the inflammatory process in FCE (57), leading to inflammatory CNV. Invernizzi et al. (58) suggested that from none CNV-AMD, or inactive CNV AMD to active CNV AMD, both choroidal thickness and CVI increased significantly, indicating the development or recurrence of CNV even before they are otherwise evident clinically. However, we found no differences between active CNV eyes and inactive CNV eyes in terms of age, SFCT, CVI, or VD, because enface OCTA images only allow moderate discrimination of CNV; furthermore, OCTA alone is inadequate for identification of active CNV requiring treatment (59). Nevertheless, our findings indicated that age, SFCT, patched hyper-reflective lesions in choriocapillaris layers, and DLSs were predictive factors for CNV progression in PSD eyes.

This study had some limitations. First, different devices were used to perform OCT and OCTA, which may have increased the potential for error. To our knowledge, the usage of different OCT devices is inevitable in real world analysis and multicenter studies with stable and reliable data. It is necessary to uniform the standard of imaging grading and measurement. Second, the sample size was small and may have limited the accuracy of the data. These limitations will be addressed in future studies.

In conclusion, this is the first study to qualitatively and quantitatively compare characteristics between atrophic and neovascular patterns, both of which were late stage lesions; to investigate the activity of CNV in PSD; and to use OCTA to examine the types of polypoidal lesions. Our findings revealed SFCT and CVI similarities between neovascular PSD eyes and atrophic PSD eyes. Additionally, we found that SFCT, age, patched hyper-reflective lesions in choriocapillaris layers, and DLSs were risk factors for CNV progression in PSD eyes. Similar to neovascular pattern, we hypothesize that atrophic PSD is an important component of PSD in the late stage, which will aid in understanding its etiology and developing appropriate treatment strategies.

## Data Availability Statement

The original contributions presented in the study are included in the article/supplementary material, further inquiries can be directed to the corresponding author.

## Ethics Statement

The studies involving human participants were reviewed and approved by Institutional Review Board of the First Hospital of China Medical University. The patients/participants provided their written informed consent to participate in this study.

## Author Contributions

RH and MZ: Conception and design, analysis and interpretation of data, writing, review, and revision of the manuscript. RH: Development of methodology, acquisition of data, administrative, technical, and material support. MZ: Study supervision. All authors contributed to the article and approved the submitted version.

## Funding

This study was funded by the Beijing Bethune Charitable Foundation [Grant No. AF-OG-03-1.1-03].

## Conflict of Interest

The authors declare that the research was conducted in the absence of any commercial or financial relationships that could be construed as a potential conflict of interest.

## Publisher's Note

All claims expressed in this article are solely those of the authors and do not necessarily represent those of their affiliated organizations, or those of the publisher, the editors and the reviewers. Any product that may be evaluated in this article, or claim that may be made by its manufacturer, is not guaranteed or endorsed by the publisher.
